# Dietary-Induced Signals That Activate the Gonadal Longevity Pathway during Development Regulate a Proteostasis Switch in *Caenorhabditis elegans* Adulthood

**DOI:** 10.3389/fnmol.2017.00254

**Published:** 2017-08-09

**Authors:** Netta Shemesh, Lana Meshnik, Nufar Shpigel, Anat Ben-Zvi

**Affiliations:** Department of Life Sciences, The National Institute for Biotechnology in the Negev Ben-Gurion University of the Negev, Beer Sheva, Israel

**Keywords:** aging, arachidonic acid (AA), *Caenorhabditis elegans*, dihomo-γ-linolenic acid (DGLA), germline stem cells (GSCs), neurodegenerative diseases, proteostasis, reproduction

## Abstract

Cell-non-autonomous signals dictate the functional state of cellular quality control systems, remodeling the ability of cells to cope with stress and maintain protein homeostasis (proteostasis). One highly regulated cell-non-autonomous switch controls proteostatic capacity in *Caenorhabditis elegans* adulthood. Signals from the reproductive system down-regulate cyto-protective pathways, unless countered by signals reporting on germline proliferation disruption. Here, we utilized dihomo-γ-linolenic acid (DGLA) that depletes the *C. elegans* germline to ask when cell-non-autonomous signals from the reproductive system determine somatic proteostasis and whether such regulation is reversible. We found that diet supplementation of DGLA resulted in the maintenance of somatic proteostasis after the onset of reproduction. DGLA-dependent proteostasis remodeling was only effective if animals were exposed to DGLA during larval development. A short exposure of 16 h during the second to fourth larval stages was sufficient and required to maintain somatic proteostasis in adulthood but not to extend lifespan. The reproductive system was required for DGLA-dependent remodeling of proteostasis in adulthood, likely via DGLA-dependent disruption of germline stem cells. However, arachidonic acid (AA), a somatic regulator of this pathway that does not require the reproductive system, presented similar regulatory timing. Finally, we showed that DGLA- and AA-supplementation led to activation of the gonadal longevity pathway but presented differential regulatory timing. Proteostasis and stress response regulators, including *hsf-1* and *daf-16*, were only activated if exposed to DGLA and AA during development, while other gonadal longevity factors did not show this regulatory timing. We propose that *C. elegans* determines its proteostatic fate during development and is committed to either reproduction, and thus present restricted proteostasis, or survival, and thus present robust proteostasis. Given the critical role of proteostatic networks in the onset and progression of many aging-related diseases, such a choice could impact susceptibility to protein misfolding diseases later in life.

## Introduction

Cellular quality control networks monitor the cellular proteome by scanning for damaged, misfolded or aggregated proteins and repairing or removing these proteins to maintain protein homeostasis (proteostasis) (Bar-Lavan et al., 2016a; Bett, 2016; Jackson and Hewitt, 2016; McCaffrey and Braakman, 2016; Voos et al., 2016). When proteostasis is breached, cells can activate stress response pathways that induce the expression of various quality control machineries to counter the flux of damaged proteins and restore proteostasis (Morimoto, 2011; Walter and Ron, 2011; Dubnikov et al., 2017; Fiorese and Haynes, 2017). However, recent studies have demonstrated that cell-non-autonomous signals can regulate these responses and inhibit or induce stress response pathways, overriding cellular proteostasis regulation and modulating the onset of protein aggregation and toxicity in models of polyglutamine (polyQ) diseases (Schinzel and Dillin, 2015; Bar-Lavan et al., 2016b; O’Brien and van Oosten-Hawle, 2016; Shemesh and Ben-Zvi, 2016). Understanding how cellular proteostasis is regulated cell-non-autonomously could thus offer novel interventions for the treatment of protein misfolding diseases.

In *Caenorhabditis elegans*, inefficient stress response activation and reduced protein folding capacity during aging are regulated cell-non-autonomously within a highly specific timeframe, beginning a few hours after the onset of reproduction. Both the basal and induced proteostatic machineries show a sharp functional decline in various somatic cells. This, in turn, results in a reduced ability of cells to protect themselves from acute stresses and chronic challenges associated with aggregation-prone proteins (Vilchez et al., 2012; Shemesh et al., 2013; Yerbury et al., 2016). For stress-induced genes, this collapse is regulated, at least in part, by a remodeling of chromatin accessibility, mediated by signals from the reproductive system (Lapierre et al., 2011; Vilchez et al., 2012; Shemesh et al., 2013; Labbadia and Morimoto, 2015b; Merkwirth et al., 2016).

Signals from the reproductive system are known to regulate organismal metabolism, stress resistance and lifespan (Hsin and Kenyon, 1999; Arantes-Oliveira et al., 2002; Flatt et al., 2008; Mason et al., 2009; Antebi, 2012; Yunger et al., 2017). Specifically, the gonadal longevity pathway mediates hormonal regulation from the somatic gonad in response to the proliferative state of the germline in worms and flies (Antebi, 2012; Amrit and Ghazi, 2017). Proliferation of germline stem cells (GSCs) results in inhibited activity of steroid hormone signaling that causes a switch from the reproductive to the survival mode at the organismal level (Wang et al., 2008; McCormick et al., 2012; Shemesh et al., 2013; Labbadia and Morimoto, 2015a). Moreover, inhibition of GSC proliferation induces transcriptional reprograming, activating a wide range of transcription factors that induce the expression of genes associated with detoxification, the quality control machinery and lipid metabolism (Hsin and Kenyon, 1999; Wang et al., 2008; O’Rourke et al., 2009; Goudeau et al., 2011; Lapierre et al., 2011, 2013; McCormick et al., 2012; Shemesh et al., 2013; Ratnappan et al., 2014; Steinbaugh et al., 2015; Amrit and Ghazi, 2017). This, in turn, negates proteostatic remodeling at the onset of reproduction (Lapierre et al., 2011; Vilchez et al., 2012; Shemesh et al., 2013; Shai et al., 2014; Labbadia and Morimoto, 2015a).

Germline stem cell-dependent lifespan extension is associated with increased production of polyunsaturated fatty acids (PUFAs), such as the ω-6 fatty acids dihomo-γ-linolenic acid (DGLA) and arachidonic acid (AA) (O’Rourke et al., 2013; Folick et al., 2015). Moreover, diet supplementation of DGLA or AA extends *C. elegans* lifespan and induces autophagy in both *C. elegans* and tissue culture models (O’Rourke et al., 2013). Given that DGLA and AA are precursors for many signaling molecules (Vrablik and Watts, 2013), these PUFAs could play a role in mediating the transfer of gonadal longevity signals to the soma. Indeed, diet supplementation of AA uncouples somatic proteostasis from the reproductive system, mimicking the proteostatic benefits of GSC arrest without negatively impacting reproduction (Shemesh et al., 2017).

DGLA-derived eicosanoid signals were recently shown to induce sterility by triggering germline depletion during development or adulthood (Watts and Browse, 2006; Webster et al., 2013; Deline et al., 2015). Here, we asked whether DGLA effectively disrupts the germline to activate the gonadal longevity pathway and could be used as a conditional regulator of this pathway and thus, of proteostasis. We found that DGLA diet supplementation resulted in the maintenance of somatic proteostasis past the onset of reproduction in such a manner that was fully dependent on the reproductive system and the gonadal signaling pathway. However, the impact of DGLA on proteostasis and stress response pathways in adulthood was set at a specific time window during development, with only a short exposure (16 h) during the second to fourth larval-stages being required and sufficient for modulating somatic proteostasis in adulthood. This regulation point is shared by the reproductive system and the soma, as a short exposure to AA during the second to fourth larval-stages was also sufficient and required to modulate proteostasis and stress activation of gonad-less animals, independent of the reproductive system. Yet, this timing is not sufficient to activate full transcriptional reprograming of the gonadal longevity pathway and thus, full lifespan extension. We, therefore, suggest that animals are committed to restricted or robust proteostatic states during development and that the choice between reproduction or survival at this point specifically determines proteostatic capacity and hence, susceptibility to protein misfolding diseases later in life.

## Results

### Dietary Supplementation of DGLA during Development Resulted in Reduced Toxicity in PolyQ-Disease Models

**FIGURE 1:**
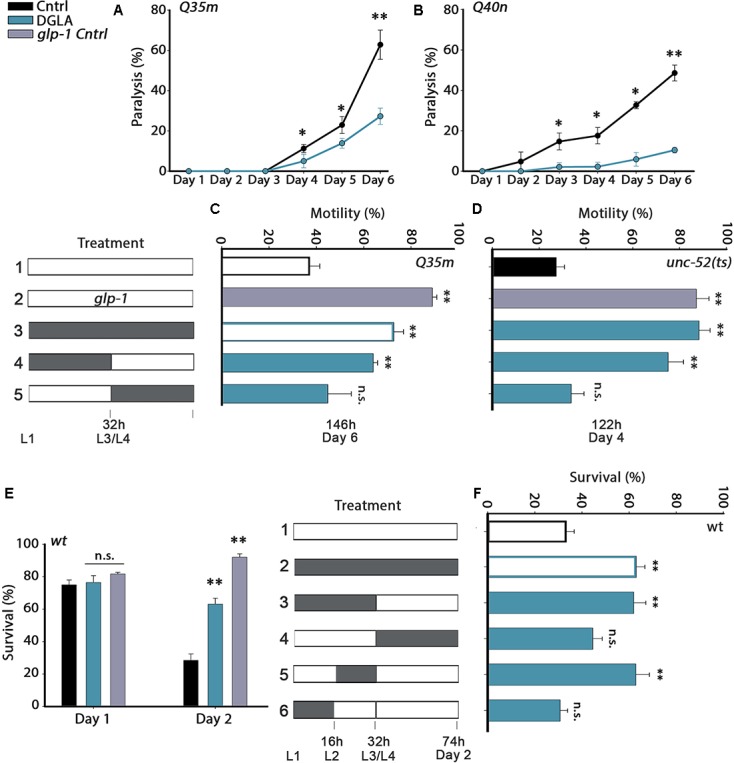
Diet supplementation of dihomo-γ-linolenic acid (DGLA) during development modulates proteostasis in adulthood. **(A,B)** Motility was scored in age-synchronized animals grown on control or DGLA-supplemented plates by determining the percentage of paralyzed **(A)** *Q35m* animals (>120 animals per data point, *N* > 5) or **(B)** *Q40n* animals (>45 animals per data point, *N* = 3) at the indicated times. **(C)** Motility was scored in age-synchronized *Q35m* or *glp-1;Q35m* animals, treated as indicated, by determining the percentage of motile animals (>45 animals per treatment, *N* > 3). **(D)** Motility was scored in age-synchronized *unc-52(ts)* or *glp-1;unc-52(ts)* animals, treated as indicated, by determining the percentage of motile animals on day 4 of adulthood (>60 animals per treatment, *N* > 4). **(E)** Thermo-resistance was examined in age-synchronized wild type or *glp-1* animals grown on control or DGLA-supplemented plates. Animals were subjected to heat shock (37°C, 6 h) at the indicated times and survival was assayed (>110 animals per treatment, *N* > 4). **(F)** Thermo-resistance was examined in age-synchronized wild type animals, treated as indicated. Animals were subjected to heat shock (37°C, 6 h) on day 2 of adulthood and survival was assayed (>120 animals per treatment, *N* > 7). Data were compared to age-matched animals grown on control plates. (n.s.) denotes non-significant, ^∗^*P* < 0.05, ^∗∗^*P* < 0.01.

Proteostasis remodeling is thought to aggravate aging-associated protein misfolding diseases. Accordingly, we first asked whether DGLA supplementation could rescue protein toxicity in *C. elegans* models of polyQ-diseases. For this, embryos expressing 35 glutamine-repeats fused to a fluorescent protein in body wall muscle (Q35m) or 40 glutamine-repeats fused to a fluorescent protein in neurons (Q40n) were transferred to control or DGLA-supplemented plates. Animals were maintained on these plates throughout the experiment and toxicity was examined during adulthood. DGLA supplementation reduced Q35m-associated age-dependent paralysis. By day 6 of adulthood, 63 ± 7% of the animals grown on control plates were paralyzed, as compared to 27 ± 4% of the Q35m animals grown on DGLA-supplemented plates (**Figure 1A**). However, only a mild effect of DGLA supplementation on the number of Q35m foci was observed (Supplementary Figure S1). Given that toxicity is not always associated with reduced aggregation and that some aggregates are protective (Cohen et al., 2006; Silva et al., 2011), this could be expected. DGLA supplementation also abrogated Q40n-associated age-dependent paralysis. While 49 ± 4% of the Q40n animals grown on control plates were paralyzed by day 6 of adulthood, only 10 ± 1% of the Q40n animals grown on DGLA-supplemented plates were paralyzed (**Figure 1B**). Thus, DGLA supplementation reduced the toxicity associated with muscular and neuronal models of polyQ-diseases in *C. elegans*.

Proteostasis remodeling occurs between days 1 and 2 of adulthood, at the onset of reproduction (Shemesh et al., 2013). We, therefore, asked when diet supplementation of DGLA is required, i.e., during development or adulthood, to rescue polyQ toxicity. As such, we shifted animals from DGLA-supplemented plates to control plates or from control to DGLA-supplemented plates at the transition to the fourth larval stage (L3/L4) and examined proteostatic functions later in adulthood. Q35m animals were grown on plates supplemented with DGLA during development (0–32 h) and then shifted to control plates for the reminder of the experiment (until day 6 of adulthood), when motility was examined. Similar to animals grown continuously on DGLA, most animals exposed to DGLA only during development were motile on day 6 of adulthood (73 ± 4% and 64 ± 2%, respectively; **Figure 1C**). DGLA-dependent rescue of motility was comparable to germline-less *glp-1(e2141)* mutant animals crossed to Q35m (Q35m; *glp-1*; 89 ± 2%). In contrast, when animals were exposed to DGLA starting from the L3/L4 transition (>32 h), motility did not improve (even after 6 days on DGLA). Only 45 ± 10% of the animals was motile, similar to animals grown continuously on control plates (37 ± 4%; **Figure 1C**). Thus, DGLA supplementation only during development was sufficient and required to reduce polyQ-associated toxicity in *C. elegans*.

### Dietary Supplementation of DGLA during Development Led to Proteostasis Rescue in Adulthood But Had No Effect on Lifespan

To extend our analysis to basal proteostatic capacity, we next asked whether DGLA supplementation during development could rescue the function of *unc-52(ts)*, an established folding reporter that shows an age-dependent decline in motility (stiff body paralysis) under permissive conditions (Ben-Zvi et al., 2009). We monitored *unc-52(ts)*-dependent paralysis on day 4 of adulthood, by which point only 27 ± 4% of the animals grown on control plates were motile. DGLA diet supplementation rescued *unc-52(ts)*-dependent paralysis (93 ± 2.5% motile animals), similar to *unc-52(ts)* animals crossed to *glp-1(e2141)* (*unc-52(ts);glp-1*; 88 ± 5% motile animals, **Figure 1D**). Moreover, motility was improved to a similar extent when *unc-52(ts)* animals were grown on DGLA-supplemented plates only during development (0–32 h; 79 ± 7%). Motility of *unc-52(ts)* animals shifted to DGLA-supplemented plates after the L3/L4 transition showed no improvement and was similar to animals grown continuously on control plates (34 ± 6%; **Figure 1D**). Thus, exposure to DGLA only during development was also sufficient and required to modulate basal proteostasis in adulthood.

We next examined whether dietary supplementation of DGLA could abrogate the decline in heat shock survival rates at the transition to reproductive adulthood. While survival rates of wild type young adults grown on control plates declined sharply between days 1 and 2 of adulthood (75 ± 3% and 28 ± 4%, respectively), survival rates of days 1 and 2 adults grown on DGLA-supplemented plates did not decline (76 ± 4% and 69 ± 2%, respectively; **Figure 1E**), similar to *glp-1(e2141)* animals (82 ± 1% and 92 ± 3%, **Figure 1E**). We then asked whether heat shock survival rates of animals grown on DGLA only during development or adulthood are comparable to the survival of animals grown on DGLA-supplemented plates over the entire duration of the experiment. Exposure to DGLA only during development (0–32 h) was as efficient as constant exposure to DGLA for rescuing heat shock survival rates of adult animals on day 2 of adulthood (63 ± 4%; **Figure 1F**). In contrast, transfer to DGLA-supplemented plates at the L3/L4 stage did not significantly rescue heat shock survival rates (44 ± 4%; **Figure 1F**). We then further restricted the time on DGLA-supplemented plates to either the first 16 h period (L1–L2) or second 16 h period (L2–L4) of the 32 h window. A short exposure to DGLA during L2–L4 but not during the L1–L2 stages was sufficient to maintain high heat shock survival rates on day 2 of adulthood (63 ± 6% and 31 ± 3%, respectively; **Figure 1F**). Thus, a short exposure to DGLA during larval development was sufficient and required to modulate heat shock survival in adulthood.

Finally, we asked whether a short exposure to DGLA during development (0–32 h) would also be sufficient to extend the lifespan of wild type animals, similar to *glp-1(e2141)* animals. Wild type animals were either exposed to DGLA during development (0–32 h) or during adulthood (transferred to DGLA after 32 h on control plates) and lifespan was compared to wild type animals maintained continuously on control or DGLA-supplemented plates at 25°C. While continuous exposure to DGLA resulted in ∼15% lifespan extension, a short exposure during development (0–32 h) or exposure to DGLA only during adulthood (starting from the L4 transition stage) resulted in a more moderate effect on lifespan (∼5–10%; Supplementary Figure S2 and Table S1). Thus, exposure to DGLA during development was not sufficient or required for DGLA to impact lifespan, supporting the hypothesis that proteostasis regulation is not necessarily associated with lifespan extension (Cohen et al., 2010; El-Ami et al., 2014).

### DGLA-Dependent Remodeling of Proteostasis Requires the Gonad

**FIGURE 2:**
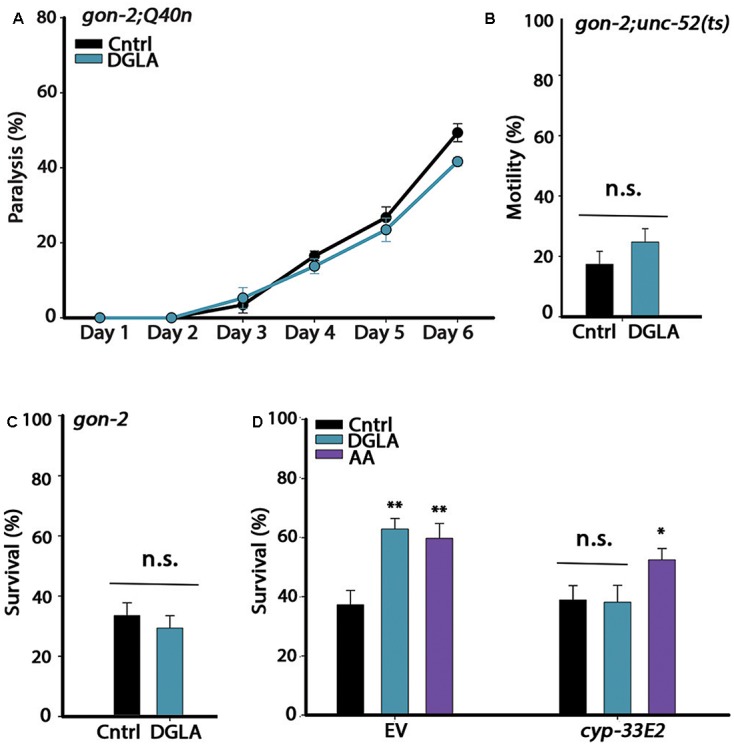
The gonad is required for DGLA-dependent proteostasis remodeling in adulthood. **(A)** Motility was scored in age-synchronized *gon-2;Q40n* animals grown on control or DGLA-supplemented plates by determining the percentages of paralyzed animals at the indicated times (>45 animals per data point, *N* > 3). **(B)** Motility was scored in age-synchronized *gon-2;unc-52(ts)* animals grown on control or DGLA-supplemented plates by scoring for stiff-body paralysis on day 4 of adulthood (>65 animals per data point, *N* > 5). **(C)** Thermo-resistance was examined in age-synchronized *gon-2(q388ts)* animals grown on control or DGLA-supplemented plates. Animals were subjected to a prolonged heat shock treatment (37°C, 6 h) on day 2 of adulthood and survival was assayed (>65 animals per data point, *N* > 5). **(D)** Thermo-resistance was examined in age-synchronized wild type animals grown on control, or DGLA- or AA-supplemented RNAi plates. Animals treated with empty vector (EV) or *cyp-32E2* RNAi, were subjected to heat shock (37°C, 6 h) on day 3 of adulthood and survival was assayed (>80 animals per data point, *N* > 5). Data were compared to age-matched animals grown on control plates. (n.s.) denotes non-significant, ^∗^*P* < 0.05, ^∗∗^*P* < 0.01.

Dietary supplementation of DGLA during development resulted in proteostasis remodeling (**Figure 1**). Because GSC arrest is associated with increased production of DGLA and DGLA-derived eicosanoids induce GSC depletion, we subsequently asked whether DGLA required the reproductive system to elicit this effect. For this, we considered whether dietary supplementation of DGLA affected the proteostasis of *gon-2(9388)* (*gon-2*) mutant animals lacking the entire reproductive system. We first crossed *gon-2* animals to Q40n (*gon-2;Q40n*) or *unc-52(ts)* (*gon-2;unc-52*) animals and compared their motility after continuous growth on control or DGLA-supplemented plates (Q35m animals could not be tested as the array was integrated close to the *gon-2* gene). While DGLA supplementation resulted in improved motility of Q40n animals (**Figure 1B**), the percent of motile *gon-2;Q40n* animals was not significantly different between animals grown on control or DGLA-supplemented plates (**Figure 2A**). Similarly, the percent of motile *gon-2;unc-52* animals on control or DGLA-supplemented plates was not significantly increased (17 ± 4% and 25 ± 4%, respectively; **Figure 2B**). Finally, DGLA supplementation improved wild type heat shock survival rates (**Figure 1E**) but could not improve survival rates of *gon-2* mutant animals when grown on control or DGLA-supplemented plates (37 ± 7% and 30 ± 6%, respectively; **Figure 2C**). As expected, supplementation of DGLA only during development (0–32 h) was as ineffective as continuous supplementation in rescuing heat shock survival rates of *gon-2(ts)* (36 ± 12% and 37 ± 4%, respectively, Supplementary Figure S3). Thus, DGLA-dependent proteostasis remodeling requires the somatic gonad.

Loss, reduced numbers or disruption of GSCs and sterility were observed in animals supplemented with DGLA either intermittently (during development or adulthood) or continuously (Watts and Browse, 2006) (Supplementary Figure S4). Given that even a short disruption to GSC proliferation during development or adulthood extends lifespan and induces GSC-dependent transcription (Arantes-Oliveira et al., 2002; Wang et al., 2008), we considered that the impact of DGLA on proteostasis could be mediated by GSC disruption. Specifically, the cytochrome P450 monooxygenase CYP-32E2 that is expressed in the pharynx was shown to produce various DGLA-derived eicosanoids that act in the gonad to induce germline defects and sterility (Deline et al., 2015). As such, we next examined whether RNAi knockdown of *cyp-33E2*, known to inhibit DGLA-dependent sterility (Deline et al., 2015), could affect DGLA-mediated rescue of heat shock survival rates. While animals grown on empty vector (EV)-containing control bacteria and DGLA-supplemented plates were able to survive heat shock treatment on day 3 of adulthood, the survival of animals grown on *cyp-33E2(RNAi)* bacteria supplemented with DGLA was similar to that of animals grown on control plates (38 ± 6% and 39 ± 6%, respectively; **Figure 2D**). The gonadal longevity pathway also elevates AA levels (O’Rourke et al., 2013; Folick et al., 2015). AA modulates proteostasis of gonad-less animals but does not affect GSC proliferation or reproduction (Shemesh et al., 2017). In agreement, survival of animals grown on *cyp-33E2(RNAi)* bacteria supplemented with AA was similar to their survival on EV control plates (53 ± 4% and 60 ± 5%, respectively; **Figure 2D**). *cyp-33E2*-mediated sterility is, therefore, specifically required for DGLA-mediated protective effects in the soma. This was supported by DGLA supplementation to germline-less *glp-1(e2141)* or *glp-4(bn2)* mutant animals that did not further improve (and even slightly reduced) heat shock survival rates on day 2 of adulthood, as compared to the survival rates of wild type animals supplemented with DGLA (Supplementary Figure S5). Thus, local DGLA-derived eicosanoids that act specifically in the gonad and disrupt GSC proliferation (Deline et al., 2015) could potentially activate the gonadal-longevity signaling pathway.

### AA Supplementation during Development Led to Remodeling of Proteostasis in Gonad-Less Animals

**FIGURE 3:**
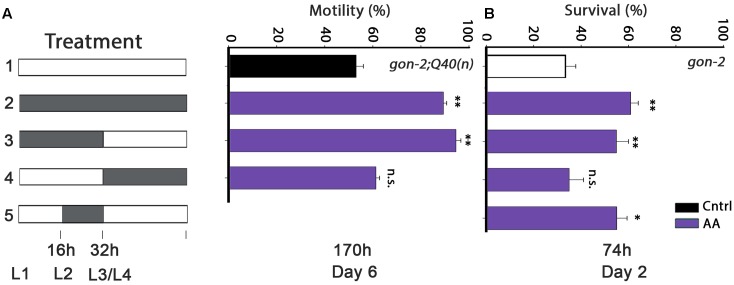
Diet supplementation of arachidonic acid (AA) during development modulates proteostasis of gonad-less animals in adulthood. **(A)** Motility was scored in age-synchronized *gon-2:Q40n* animals, treated as indicated, by determining the percentage of motile animals (>60 animals per treatment, *N* > 4). **(B)** Thermo-resistance was examined in age-synchronized *gon-2(q388ts)* animals, treated as indicated. Animals were subjected to a prolonged heat shock treatment (37°C, 6 h) on day 2 of adulthood and survival was assayed (>65 animals per data point, *N* > 5). Data were compared to age-matched animals grown on control plates. (n.s.) denotes non-significant, ^∗^*P* < 0.05, ^∗∗^*P* < 0.01.

The timing of DGLA requirement could simply depend on the ability of DGLA to eliminate GSCs. Although, under our conditions, DGLA supplementation only induced partial sterility and reduced brood size (Supplementary Figure S4), addition of DGLA at the L2–L4 larval stages is most effective to eliminate GSCs (Watts and Browse, 2006). As noted above, AA also modulates proteostasis in adulthood, in a manner independent of the reproductive system (Shemesh et al., 2017). To examine whether the L2–L4 window is associated with GSC disruption, we next examined the timing of AA requirement for proteostasis remodeling in wild type or gonad-less animals. AA diet supplementation rescued *gon-2;Q40n*-dependent paralysis (89 ± 1.5% motile animals by day 6 of adulthood) and motility was improved to a similar extent when *gon-2;Q40n* animals were grown on AA-supplemented plates only during development (0–32 h; 94 ± 2%; **Figure 3A**). In contrast, motility of *gon-2;Q40n* animals shifted to AA-supplemented plates after the L3/L4 transition was similar to animals grown continuously on control plates (61 ± 3% and 53 ± 3%, respectively; **Figure 3A**). AA supplementation to *gon-2* animals only during development (0–32 h), specifically in the 16–32 h window, was also sufficient to modulate heat shock survival rates in adulthood (55 ± 5% and 55 ± 4%, respectively; **Figure 3B**), similar to the wild type (50 ± 5% and 49 ± 5%, respectively; Supplementary Figure S6). In contrast, AA supplementation starting from L3/L4 transition of either wild type or *gon-2* animals had no effect on survival (35 ± 2% and 35 ± 6%, respectively; **Figure 3B** and Supplementary Figure S6). Thus, our data demonstrate that the L2–L4 period is critical for determining proteostatic capacity during adulthood even in gonad-less animals, suggesting that this period is detrimental to proteostasis in adulthood of both the soma and the reproductive system. However, this timing was not required for lifespan extension, similar to DGLA (Supplementary Table S1).

### DGLA- and AA-Supplementation Led to Remodeling of the Heat Shock Response Switch at the Onset of Reproduction

**FIGURE 4:**
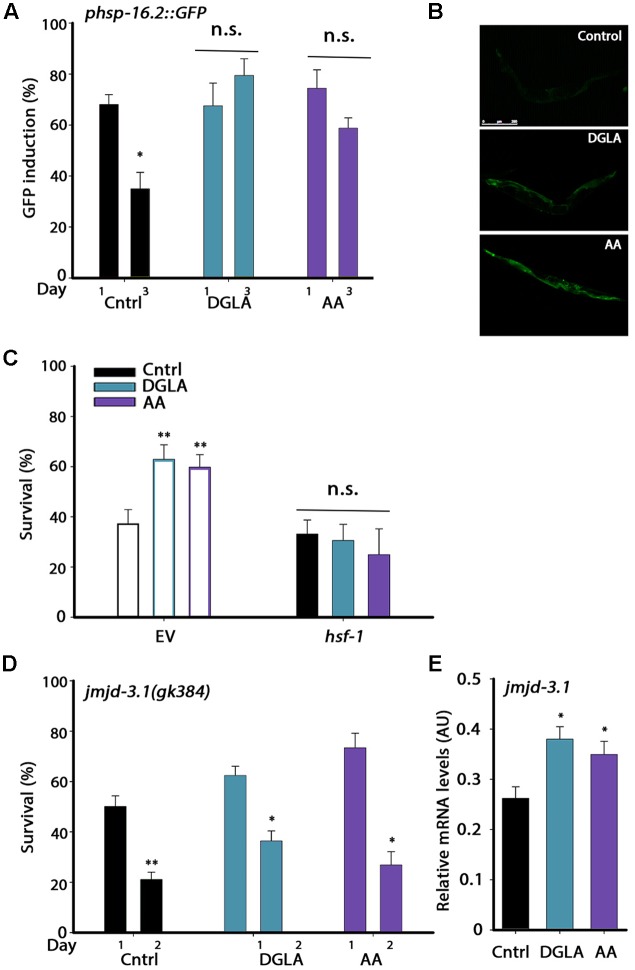
DGLA and AA supplementation results in remodeling of the heat shock response switch in adulthood. **(A)** Heat shock gene induction was examined in age-synchronized animals expressing *phsp-16.2*::*GFP* grown on control, DGLA- or AA-supplemented plates. Animals were subjected to heat shock (37°C, 90 min) at the indicated times and fluorescence was monitored after a 24 h recovery period. Animals expressing GFP in somatic tissues (other than the pharynx) were scored (>60 animals per treatment, *N* > 3). **(B)** Representative images of day 3 adult animals expressing *phsp-16.2*::*GFP* grown on control, or DGLA- or AA-supplemented plates as in A. **(C)** Thermo-resistance was examined in age-synchronized wild type animals grown on control, or DGLA- or AA-supplemented RNAi plates. Animals treated with EV or *hsf-1* RNAi were subjected to heat shock (37°C, 6 h) on day 3 of adulthood and survival was examined (>70 animals per treatment, *N* > 4). **(D)** Thermo-resistance was examined in age-synchronized *jmjd-3.1(gk384)* animals grown on control, or DGLA- or AA-supplemented plates. Animals were subjected to heat shock treatment (37°C, 6 h) at the indicted times and survival was examined (>130 animals per treatment, *N* > 5). **(E)** Quantification of *jmjd-3.1* mRNA levels from age-synchronized animals grown on control, or DGLA- or AA-supplemented plates (*N* > 5). For **(A,D)** data were compared to similarly treated animals on day 1 of adulthood. For **(C,E)**, data were compared to age-matched animals grown on control plates. (n.s.) denotes non-significant, ^∗^*P* < 0.05, ^∗∗^*P* < 0.01.

Having established that the L2–L4 period is critical for proteostasis remodeling but not for lifespan extension, we next asked whether DGLA and AA supplementation resulted in the activation of the different stress and metabolic transcription factors associated with the gonadal longevity pathway and if so, when. We first examined whether DGLA and AA supplementation remodeled the heat shock response switch regulated by the gonadal longevity pathway at the onset of reproduction. Exposing animals to stress even 6 h after the onset of reproduction results in limited induction of cyto-protective heat shock genes required to confer resistance to the stress. The decline in response activation is associated with chromatin remodeling and is reflected in reduced levels of heat shock gene induction (Shemesh et al., 2013; Labbadia and Morimoto, 2015b). To examine whether DGLA and AA supplementation led to modulation of this switch, as in the gonadal longevity pathway, the induction of heat shock genes was examined. For this, *hsp-16.2*-regulated green fluorescent protein (GFP) that served as a reporter expressed in different somatic tissues upon heat shock was monitored. GFP-regulated expression was not significantly different between days 1 and 3 of adulthood in animals grown on DGLA-supplemented plates (68 ± 9% and 79 ± 7%, respectively) or AA-supplemented plates (71 ± 6% and 59 ± 4%, respectively) but declined sharply in control-treated animals (68 ± 4% and 36 ± 7%, respectively; **Figures 4A,B**). Likewise, *hsp-70* and *F44E5.4* induction, examined using qPCR, was two–four fold lower on day 2, as compared to day 1 adults grown on control plates, yet remained high when animals were grown on DGLA-supplemented plates (Supplementary Figure S7). Thus, dietary supplementation of DGLA or AA can negate the decline in heat shock activation at the onset of reproduction.

The methyltransferase JMJD-3.1 is required for HSF-1-dependent heat shock response activation and expression of heat shock genes in the soma after reproduction onset. *jmjd-3.1* expression is reduced at the transition to reproductive adulthood and its increased expression is associated with GSC-dependent rescue of proteostasis remodeling (Labbadia and Morimoto, 2015b). We, therefore, examined whether HSF-1 and JMJD-3.1 are required for DGLA- and AA-mediated rescue of heat shock survival. Animals were grown on EV- or *hsf-1* RNAi-expressing bacteria with or without dietary supplementation of DGLA or AA and heat shock survival was examined on day 3 of adulthood. While ∼60% of day 3 adults grown on EV bacteria supplemented with DGLA or AA survived the stress, growth on *hsf-1(RNAi)* abolished DGLA- and AA-associated beneficial effects (31 ± 6%, 25 ± 10%, respectively), reducing survival to control levels (33 ± 5%; **Figure 4C**). HSF-1 is thus required for DGLA- and AA-dependent rescue of heat shock survival. We next examined heat shock survival rates in a *jmjd-3.1(gk384)* mutant. Mutant *jmjd-3.1(gk384)* animals grown on plates supplemented with DGLA or AA showed a ∼2-fold decline in survival rates between days 1 and 2 of adulthood (62 ± 4% to 36 ± 4% and 73 ± 6% to 27 ± 5%, respectively), similar to animals grown on control plates (50 ± 4% and 23 ± 3.5%, respectively; **Figure 4D**). In agreement, *jmjd-3.1* levels, examined using qPCR, were significantly higher (∼1.4-fold) in animals grown on DGLA or AA than in animals grown on control plates (**Figure 4E**). These data suggest that dietary supplementation of DGLA or AA abrogates the decline in heat shock activation in adulthood by remodeling the JMJD-3.1- and HSF-1-regulated switch and thus, the ability to induce heat shock gene expression, similar to the gonadal longevity pathway.

### DGLA- and AA-Supplementation Resulted in the Induction of Transcriptional Reprograming Associated with the Gonadal-Longevity Pathway

**FIGURE 5:**
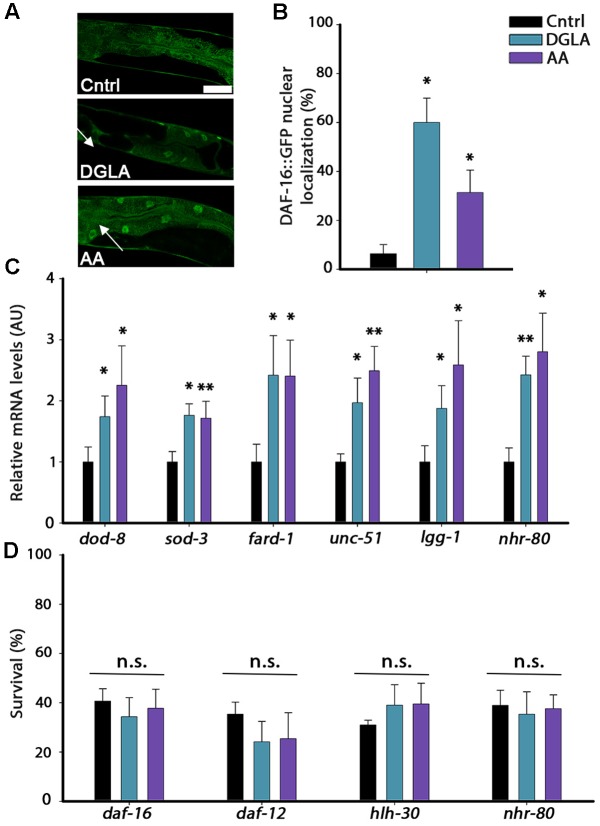
DGLA and AA supplementation results in the activation of the gonadal longevity pathway. **(A)** Representative images of age-synchronized animals expressing *DAF-16::GFP* grown on control, or DGLA- or AA-supplemented plates. Animals were fixed and imaged on day 2 of adulthood. **(B)** Age-synchronized *DAF-16::GFP* animals were grown on control, or DGLA- or AA-supplemented plates, as indicated. Animals were fixed on day 2 of adulthood and the percentage of animals showing nuclear localization of DAF-16::GFP (arrows) was scored (>400 cells per treatment, *N* > 3). **(C)** Quantification of mRNA levels of *dod-8*, *sod-1, fard-1*, *unc-51*, *lgg-1*, or *nhr-80* from age-synchronized animals grown on control, or DGLA- or AA-supplemented plates. Data were normalized to those obtained with controls (*N* > 4). **(D)** Thermo-resistance was examined in age-synchronized mutant, *daf-16*, *daf-12*, *hlh-30*, or *nhr-80* animals, grown on control, or DGLA- or AA-supplemented plates. Animals were subjected to heat shock (37°C, 6 h) on day 2 of adulthood and survival was examined (>65 animals per treatment, *N* > 3). Data were compared to age-matched animals grown on control plates. (n.s.) denotes non-significant, ^∗^*P* < 0.05, ^∗∗^*P* < 0.01.

DGLA and AA levels are elevated by overexpression of *lipl-4* that is regulated by the gonadal-longevity signaling pathway and specifically, DAF-16 (O’Rourke et al., 2013; Folick et al., 2015). We thus asked whether the gonadal longevity pathway could, in turn, be activated in DGLA- or AA-supplemented animals. Activation of the gonadal longevity pathway triggers the nuclear localization of DAF-16 in the intestine (Berman and Kenyon, 2006). To examine whether DAF-16 is activated by DGLA or AA supplementation, we monitored the localization of DAF-16 tagged with GFP (DAF-16::GFP). Animals expressing DAF-16::GFP were grown on control, DGLA- or AA-supplemented plates and DAF-16 localization was monitored. While DAF-16::GFP was equally localized in the cytoplasm and nuclei of animals grown on control plates, DGLA or AA supplementation enhanced DAF-16::GFP nuclear localization in the intestine of animals grown on DGLA- or AA-supplemented plates (6+4%, 60 ± 10%, and 31 ± 9%, respectively; **Figures 5A,B**). DGLA- and AA-dependent changes in DAF-16::GFP localization were associated with increased expression of genes regulated by DAF-16. *dod-8* and *sod-3* mRNA levels increased ∼2-fold in animals supplemented with DGLA or AA (**Figure 5C**). Moreover, diet supplementation of DGLA or AA to animals harboring a mutation in the gene encoding DAF-16/FOXO did not increase their heat shock survival (34 ± 8% or 38 ± 8, respectively; **Figure 5D**). These data suggest that DGLA and AA levels are not only regulated by the gonadal longevity pathway (O’Rourke et al., 2013; Folick et al., 2015) but can also induce DAF-16 nuclear localization and expression associated with activation of this pathway.

The gonadal longevity pathway is also associated with the activation of other transcription factors, including the steroid nuclear receptor DAF-12 and the lipid metabolism, lipolysis and autophagy transcription regulators PHA-4, HLH-30, and NHR-80 (Amrit and Ghazi, 2017). The mRNA levels of *fard-1* regulated by DAF-12, of *unc-51* and *lgg-1* regulated by PHA-4 and HLH-30 and of *nhr-80* regulated by NHR-80 (Goudeau et al., 2011; Lapierre et al., 2011, 2013; McCormick et al., 2012) were, therefore, examined using qPCR. The mRNA levels of these genes were elevated by two–three fold in animals grown on DGLA- or AA-supplemented plates, compared to controls (**Figure 5C**). In agreement, diet supplementation of DGLA or AA to animals harboring mutations in *daf-12*, *hlh-30*, or *nhr-80* did not increase their heat shock survival (**Figure 5D**). Thus, various transcription factors that are regulated by the gonadal longevity pathway are activated by exposure to DGLA or AA and are required for DGLA- or AA-dependent effects on proteostasis remodeling in adulthood. These data suggest the continuous exposure to DGLA and AA activate the gonadal longevity pathway.

### DGLA- and AA-Supplementation during Development Is Sufficient to Activate Proteostasis-Associated Pathways

**FIGURE 6:**
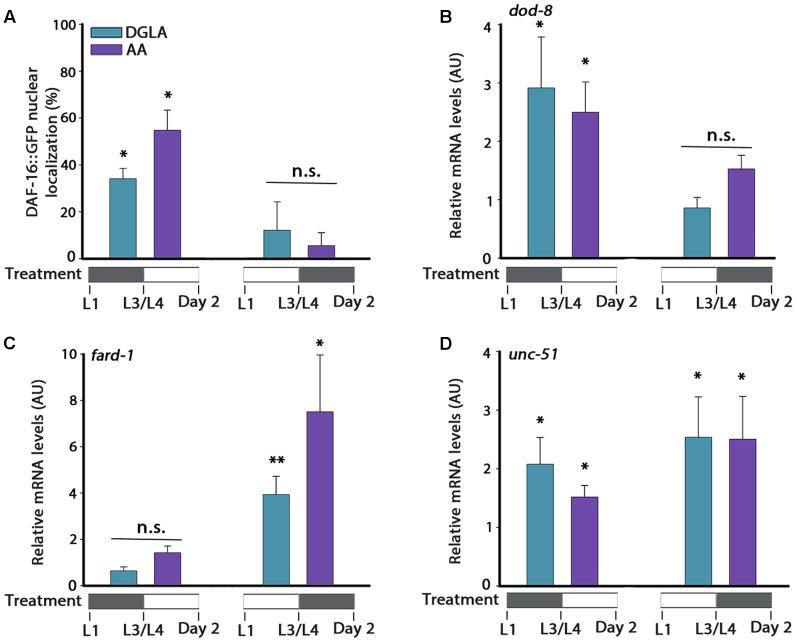
DGLA and AA supplementation during development results in partial activation of the gonadal longevity pathway. **(A)** Age-synchronized *DAF-16::GFP* animals, treated as indicated, were fixed on day 2 of adulthood and the percentage of animals showing nuclear localization of DAF-16::GFP was scored (>400 cells per treatment, *N* > 3). **(B–D)** Quantification of *dod-8* **(B)**, *fard-1* **(C)**, or *unc-51* **(D)** mRNA levels from age-synchronized animals, treated as indicated. Data were normalized to those obtained with controls (*N* > 4). Data were compared to age-matched animals grown on control plates. (n.s.) denotes non-significant, ^∗^*P* < 0.05, ^∗∗^*P* < 0.01.

We next asked whether DGLA or AA supplementation during development was sufficient to activate the different factors associated with the gonadal longevity pathway or whether it was specific to only some of factors in this pathway. Accordingly, animals were either grown on DGLA- or AA-supplemented plates during development (until L3/L4 transition) and then shifted to control plates, or first grown on control plates (until L3/L4 transition) and then shifted to DGLA- or AA-supplemented plates. In either growth protocol, gene regulation was examined. First, DAF-16 nuclear localization was examined. DGLA- or AA-supplementation before L3/L4 transition was sufficient to induce DAF-16::GFP nuclear localization in adulthood (34 ± 4% and 55 ± 9%, respectively), while supplementation of DGLA or AA starting at L3/L4 was not (12 ± 12% and 5 ± 5%, respectively; **Figure 6A**). In agreement, the expression of *dod-8* and *sod-3* was induced two–three fold by DGLA or AA supplementation before L3/L4 transition but not after (**Figure 6B** and Supplementary Figure S8). Thus, proteostasis regulation pathways linked to the gonadal longevity pathway were specifically activated early in a development.

The expression of genes regulated by other gonadal longevity pathways, such as *daf-12*, *hlh-30*, and *nhr-80*, showed different regulation behavior in time (**Figures 6C,D** and Supplementary Figure S8). Whereas expression levels of *fard-1* were not significantly induced by supplementation of DGLA or AA during development, they were induced by DGLA or AA supplementation after L3/L4 transition. On the other hand, expression levels of *unc-51* were induced regardless of the timing of DGLA or AA supplementation (**Figures 6C,D**). These data suggest that early signals can determine somatic proteostatic capacity in adulthood, although the timing of DGLA or AA supplementation differs for other factors in the gonadal longevity pathway. We, therefore, propose that the L2–L4 period is specific for proteostasis remodeling in adulthood but that other pathways, and thus lifespan, could be differentially regulated.

## Discussion

**FIGURE 7:**
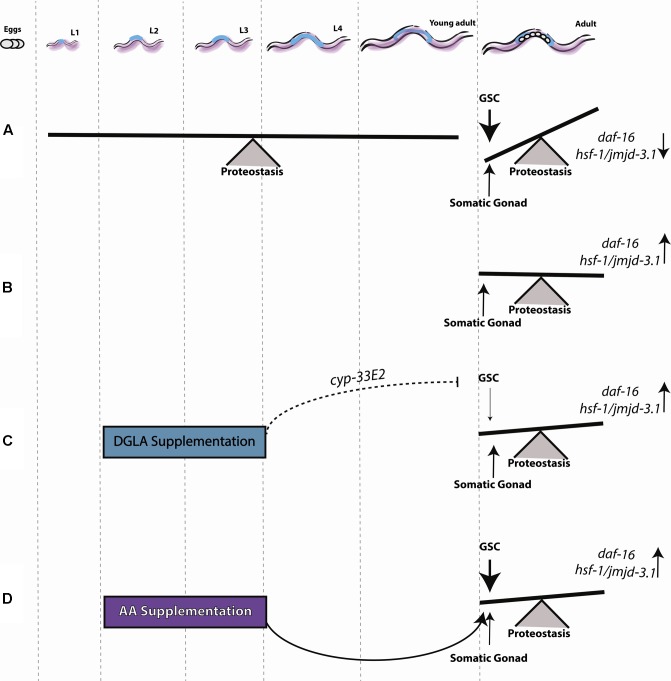
Early signals from the reproductive system determine somatic maintenance. **(A)** Proteostasis of young adult animals is robust, showing low levels of protein damage, effective activation of stress responses and high rates of stress survival. Following the onset of reproduction, signals from GSCs activate a regulatory switch that changes the proteostatic capacity of the soma. **(B)** Inhibiting GSC proliferation induces sterility and allows the organism to maintain robust proteostasis by somatic gonad signals, as seen in young animals. **(C)** DGLA supplementation during the L2–L4 stage has a beneficial effect on proteostatic collapse, depending on somatic gonad function, and a negative effect on fecundity. **(D)** AA supplementation during the L2–L4 stage has a beneficial effect on proteostasis during adulthood.

Proteostasis remodeling at the onset of *C. elegans* reproduction is a highly regulated switch, activated by signals from the reproductive system that result in the inability of somatic tissues to maintain proteostasis (**Figure 7A**). GSC arrest mitigates this response, activating the gonadal longevity pathway and maintaining juvenile proteostasis (**Figure 7B**). To understand when the fate of proteostasis is set and whether it is reversible, we employed DGLA, known to disrupt GSCs (Watts and Browse, 2006; Deline et al., 2015) and associated with extended lifespan (O’Rourke et al., 2013; Folick et al., 2015), as a conditional agent to remodel proteostasis. We found that diet supplementation of DGLA rescued the maintenance of proteostasis past the onset of reproduction only if supplemented during development. This rescue required the gonad and different effectors in the gonadal longevity pathway. Specifically, for the heat shock response, this rescue required DAF-16, HSF-1, and JMJD-3.1, suggesting that DGLA is a modulator of the proteostatic switch early in adulthood (**Figure 7C**). Although AA rescued proteostatic function in the soma, like DGLA, it was only effective when applied during development so as to rescue proteostasis in adulthood (**Figure 7D**). We, therefore, propose that proteostasis in adulthood is set during development. This regulation timing, however, was specific for proteostasis and stress response pathways. Other transcription factors that are regulated by the gonadal longevity signaling, such as metabolic and lipolysis regulators, were not specifically activated during development.

### Temporal Resolution of the Gonadal Longevity Pathway

The gonadal longevity pathway responds to GSC status by regulating a number transcription factors required for detoxification, quality control and lipid metabolism (Hsin and Kenyon, 1999; Wang et al., 2008; O’Rourke et al., 2009; Lapierre et al., 2011, 2013; Goudeau et al., 2011; McCormick et al., 2012; Shemesh et al., 2013; Ratnappan et al., 2014; Steinbaugh et al., 2015; Amrit and Ghazi, 2017). Our data demonstrate that continuous supplementation of DGLA or AA required key transcription factors that are an integral part of this pathway, such as HSF-1/JMJD-3.1, DAF-16, DAF-12, NHR-80, and HLH-30, and induced their transcriptional activities. However, our data also indicate that the timing of activation of these transcription factors differs. While supplementation of DGLA or AA during the L2–L4 stages led to remodeling of the heat shock response and the activation of HSF-1 and DAF-16, it was not required for the transcriptional activation of other transcription factors. For example, *unc-51* expression that is regulated by PHA-4 and HLH-30 was also induced when DGLA and AA were supplemented only after L4 (O’Rourke et al., 2009) and was not restricted to development (**Figure 6D**). Differential transcriptional activation is likely to be a feature of GSC signaling as Nile Red staining, indicative of lysosome-related organelles, is regulated by GSC arrest in development and adulthood (Wang et al., 2008; O’Rourke et al., 2013). Temporal differences in transcriptional activation are also evident in lifespan regulation. Arantes-Oliveira et al. (2002) specifically examined the timing of GSC-dependent regulation of lifespan and showed that GSC arrest starting from L4 stage could modulate lifespan in adulthood but that this timing resulted in a milder effect than starting during larval development. In agreement, DGLA and AA supplementation starting from L4 can extend lifespan (O’Rourke et al., 2013) but has a milder effect than continuous exposure during both development and adulthood (Supplementary Figure S2 and Table S1). We, therefore, propose that protesotasis regulation during larval development can extend lifespan but other transcription factors, such as DAF-12, PHA-4, and HLH-30 that regulate lipid metabolism and lipolysis (Lapierre et al., 2011, 2013; O’Rourke and Ruvkun, 2013), can be activated during adulthood to promote longevity.

One implication of this work is that cell-non-autonomous signals from the reproductive system cannot be employed to reverse proteostatic collapse in adult animals unless triggered during development, at least in *C. elegans*. Restoring proteostasis by modulating the gonadal longevity pathway as a possible intervention in protein misfolding diseases is, therefore, not likely. However, other cell-non-autonomous signaling pathways, including other gonadal longevity pathway-regulated transcription factors, could serve such a function (Dillin et al., 2002a; Cohen et al., 2010).

### DGLA-Mediated Actions Require the Reproductive System

The combination of signals from GSCs and the gonad were suggested to report on reproductive potential to the soma and thus modulate somatic survival and proteostatic capacity, accordingly (Shemesh et al., 2013). Our data suggest that DGLA supplementation causes GSC disruption, since: (1) DGLA-mediated rescue of somatic proteostasis required the gonad and could not further improve rescue in GSC-arrested animals (**Figure 2** and Supplementary Figures S3, S5); (2) DGLA impact required the gonadal longevity pathway. DGLA supplementation resulted in DAF-16 nuclear localization in the soma, transcriptional activation of the gonadal signaling pathway and mutant animals that were unable to activate the gonadal signaling pathway were unaffected by DGLA supplementation (**Figures 5**, **6** and Supplementary Figure 8); (3) DGLA-dependent rescue of proteostasis required CYP-33E2 (**Figure 2D**), shown to locally produce DGLA-derived eicosanoids that harm GSCs (Deline et al., 2015); and (4) the L2-L4 window that was sufficient for full somatic rescue of proteostasis (**Figure 1**) was also sufficient to disrupt reproduction (Watts and Browse, 2006) (Supplementary Figure S4). When these observations are taken together, they lead us to propose that DGLA likely acts within the reproduction system by disrupting GSC, placing it upstream to the GSCs. However, a short supplementation of AA during development was also sufficient to improve proteostasis in adulthood (**Figure 3** and Supplementary Figure S6), even though AA acts in the soma (Shemesh et al., 2017). Given that AA is also required in the gonadal longevity pathway to modulate proteostatic capacity during adulthood, it would appear that both DGLA and AA are associated with mediating signals received from the reproductive system.

### A Developmental Regulatory Checkpoint Sets Proteostatic Collapse

The second and third larval stages are critical periods in the development of the reproduction system and GSCs (Killian and Hubbard, 2005). During this window, GSCs proliferate in the gonad arm (Killian and Hubbard, 2005) and their proliferation depends on interactions with the somatic gonad (McCarter et al., 1997). Moreover, when GSC proliferation is inhibited, the somatic gonad activates the steroid nuclear receptor DAF-12 (Hsin and Kenyon, 1999; Yamawaki et al., 2010; Shen et al., 2012). Of note, DAF-12 functions in the heterochronic pathway, which controls larval development timing. Specifically, DAF-12 is involved in L2–L3 transition and this role is associated with GSC-dependent regulation of lifespan (Antebi, 2012; Shen et al., 2012). The fact that DAF-12 function coincides with the timing of DGLA and AA commitment suggests that DAF-12 function during this phase could be critical for somatic maintenance in adulthood. However, the expression of *fard-1*, regulated by DAF-12, was not induced upon DGLA or AA supplementation when animals were treated only during development (**Figure 6C**).

There are other checkpoints that determine whether or not conditions are suitable for reproduction, depending on environmental conditions and food availability (Dillin et al., 2002b; Volovik et al., 2012; Levi-Ferber et al., 2014; Schindler et al., 2014; Thondamal et al., 2014; Wang et al., 2017). One checkpoint that impacts the reproduction system is the L3 stage, when food deprivation can result in arrested development of the reproductive system. This signal is associated with *daf-16* and *daf-9* (Schindler et al., 2014) that also function in the gonadal longevity pathway (Hsin and Kenyon, 1999; Gerisch et al., 2007; Antebi, 2012). Moreover, the L3 stage is also a metabolic checkpoint, impacted by cell-non-autonomous signaling from neurons responding to mitochondrial function (Durieux et al., 2011; Merkwirth et al., 2016) or GSCs (Wei and Kenyon, 2016). In turn, disruption of mitochondrial function could also cause larval arrest at L3 (Tsang et al., 2001; Tsang and Lemire, 2003). We, therefore, propose that a balance of environmental conditions, metabolic state and reproduction potential are weighed in the L2–L3 window to determine the fate of the organism in terms of its ability to reproduce or to maintain proteostasis in the soma.

## Materials and Methods

### Nematodes and Growth Conditions

Nematodes were grown on NGM plates seeded with the *Escherichia coli* OP50-1 strain. Diet-supplemented fatty acids were prepared as previously described (Deline et al., 2013). Plates contained the detergent Tergitol (0.1%, NP-40; Sigma) were used as control or were supplemented with DGLA (90 μM; Santa Cruz Biotechnology, dissolved in Tergitol) or AA (60 μM; TCI Chemical, dissolved in Tergitol). Unless otherwise stated, 30–80 embryos, laid at 15°C, were transferred to the indicated plates (control, DGLA, or AA) and grown at 25°C for the duration of an experiment. The first day of adulthood (day 1) was set before the onset of egg-laying (50 h after temperature shift). To avoid progeny contamination, animals were moved to new plates during the reproductive period.

### Statistical Analysis

Experiments were repeated at least three times and >15 animals per experimental condition were scored. Data are presented as means ± SEM. *P*-values were calculated using the Wilcoxon Mann–Whitney rank sum test to compare two independent populations. (n.s.) denotes non-significant (^∗^) denotes *P* < 0.05, (^∗∗^) denotes *P* < 0.01. OASIS2, an online application for survival analysis (Han et al., 2016) was used to analyze lifespan assays (data included in Supplementary Figure S2 and Table S1). Mean life spans were calculated using Kaplan–Meier survival curves and *P*-values were determined using log-rank (Mantel-Cox) statistics.

### Aggregates Quantification

Age-synchronized animals (*n* > 50) that express *punc-54::Q35::YFP* (Q35m) were imaged using a Leica M165 FC fluorescent stereoscope with a YFP filter and the number of aggregates was counted. Aggregates were defined as discrete structures that are brighter and clearly distinguished from the surrounding fluorescence (bright foci) (Morley et al., 2002).

### Paralysis Assay

Age-synchronized animals (*n* > 30) were moved every day, and animals were scored by monitoring their movement 5 min after transfer to a new plate. Animals that did not move were scored as paralyzed (Karady et al., 2013).

### Stiff Body Paralysis Assay

Age-synchronized (*n* > 30) *unc-52(ts)* mutant animals were grown at 25°C until day 1 of adulthood. Animals were then shifted to 15°C and paralysis was scored on day 4 of adulthood.

### Thermo-Resistance Assay

Animals were moved to a 24-well plate containing heat shock buffer (100 mM Tris-HCl, pH 7.4, 17 mM NaCl and 1% cholesterol supplemented with bacteria) at the indicated ages and subjected to a 37°C heat shock for 6 h. Animal survival was scored by monitoring uptake of SYTOX orange (Invitrogen) dye supplemented to the buffer following heat shock, using a Leica M165 FC fluorescent stereoscope with a TXR filter. Fluorescent animals were scored as dead (Karady et al., 2013). Heat shock-treated animals were discarded after scoring.

### Lifespan Analysis

Synchronized embryos [N2 or *glp-1(e2141)*], laid at 15°C, were transferred to the indicated plates (control, DGLA or AA) and maintained at 25°C for the duration of an experiment. At L4 (after 32 h), animals were shifted to new plates (15–30 animals per plate), as indicated (control, DGLA or AA). For each treatment condition, ∼50–200 L4 animals were monitored. During the reproductive period, animals were transferred to freshly seeded plates every day. Animals were considered dead when they no longer responded to gentle prodding with a platinum wire. Scoring was performed every day. Animals that crawled off the plate, were contaminated or displayed a bagging phenotype (matricide due to internal hatching of embryos) were censored.

### DAPI Staining

Gonads were dissected as described (Colaiacovo et al., 2003), fixed and stained as previously described (Karady et al., 2013) and subsequently mounted and imaged using an Olympus Fluoview FV1000 confocal microscope through a 60× 1.0 numerical aperture objective with a 405 nm line for excitation.

### Sterility

Age-synchronized animals (*n* > 30) were grown on control or DGLA- or AA-supplemented plates at 25°C were imaged using a Leica M165 FC fluorescent stereoscope and examined for the presence of oocytes and embryo in the uterus.

### Progeny Quantification

Age-synchronized animals of all tested condition (in parallel) were allowed to lay eggs on fresh plates at 25°C. Individual animals were set of separate plates and moved every 24 h during the first 5 days of adulthood (i.e., past the reproduction span). The number of offspring was scored 48–72 h later and the progeny of >15 animals per condition were scored.

### RNA Interference (RNAi)

Embryos (*n* > 15) were placed on *E. coli* strain HT115(DE3) transformed with the indicated RNAi vectors (obtained from the Ahringer or Vidal RNAi libraries) or the empty vector (pL4440), as previously described (Shemesh et al., 2013).

### DAF-16 Nuclear Localization Assay

Age-synchronized DAF-16::GFP animals (*n* > 20) were grown on control or DGLA- or AA-supplemented plates at 25°C. Animals were fixed on day 2 of adulthood and mounted as previously described. DAF-16 nuclear localization in intestinal cells was imaged using a Leica DM5500 confocal microscope through a 40× 1.0 numerical aperture objective with a 488 nm line for excitation. Animals were scored as having nuclear-localized DAF-16 when the majority of their intestinal cells showed GFP accumulation in the nucleus (Berman and Kenyon, 2006). Animals were scored blind.

### Heat Shock Treatment

Age-synchronized animals (*n* > 30) were placed in a 37°C bath for 90 min in sealed plates. Following treatment, the animals were frozen or fixed immediately.

### RNA Levels

Forty to sixty animals were collected per condition. RNA was extracted using the TRIzol reagent (Invitrogen). mRNA was reverse-transcribed using the iScript cDNA Synthesis Kit (Bio-Rad) for cDNA synthesis. Quantitative PCR was performed on a C1000 Thermal Cycler (Bio-Rad) with KAPA SYBER FAST (KAPA BIOSYSTEMS) (Shemesh et al., 2013).

## Author Contributions

NeS and AB-Z designed the experiments. NeS, LM, and NuS performed the experiments, analyzed the data and interpreted the results. NeS and AB-Z wrote the manuscript.

## Conflict of Interest Statement

The authors declare that the research was conducted in the absence of any commercial or financial relationships that could be construed as a potential conflict of interest.
