# Supplementary material for: Dietary-Induced Signals That Activate the Gonadal Longevity Pathway during Development Regulate a Proteostasis Switch in Caenorhabditis elegans Adulthood
Source: Front Mol Neurosci. 2017 Aug 9;10:254. doi: 10.3389/fnmol.2017.00254 (PMC5552676; doi:10.3389/fnmol.2017.00254)
Supplement: Supplementary file 1 [file Presentation_1.pdf]

Supplementary Material

Dietary-induced signals that activate the gonadal longevity pathway during development regulate a proteostasis switch in *Caenorhabditis elegans* adulthood

Netta Shemesh[1], Lana Meshnik[1^], Nufar Shpigel[1^] and Anat Ben-Zvi[1*]

[1]The National Institute for Biotechnology in the Negev, Department of Life Sciences, Ben-Gurion University of the Negev, Beer Sheva 84105, Israel

[^]These authors contributed equally to this work.

*** Correspondence:**
Anat Ben-Zvi
anatbz@bgu.ac.il

1 Supplementary Figures

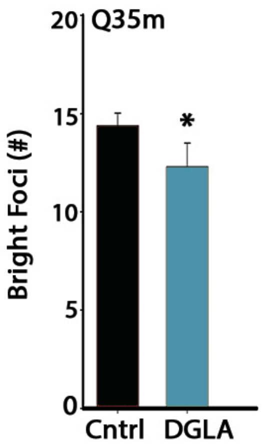

Supplementary Figure 1. Diet supplementation of DGLA does not modulate polyQ aggregation. The number of bright foci scored on day 2 of adulthood in age-synchronized *Q35m* animals grown on control or DGLA-supplemented plates (>40 animals per data point, N>3). (*) denotes $P<0.05$.

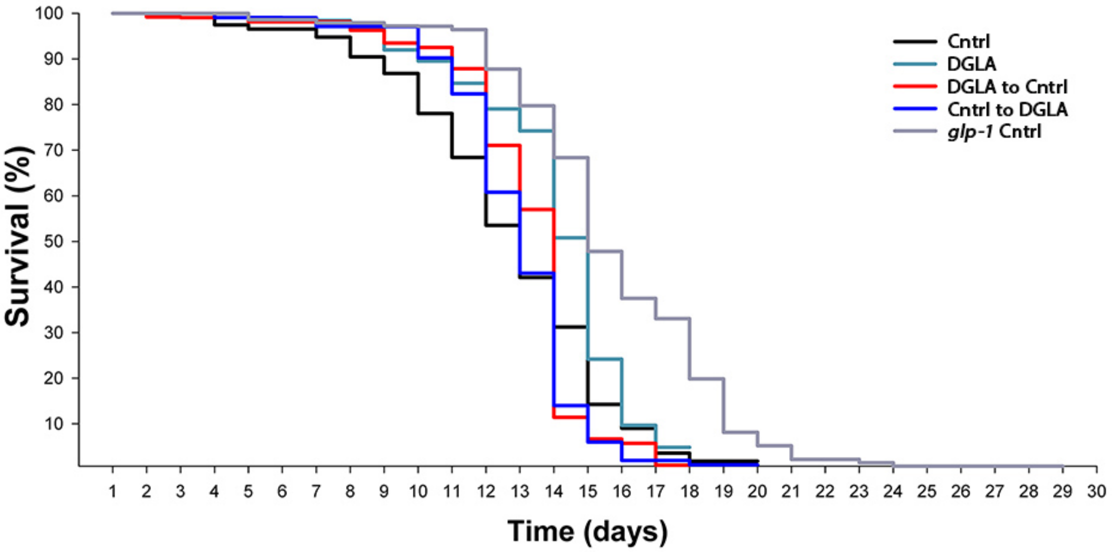

Supplementary Figure 2. Effects of DGLA supplementation on lifespan. Lifespan of wild type (N2) or *glp-1* animals grown on control or DGLA-supplemented plates, continuously or shifted between treatment and control at the L3/L4 transition (>32 h). Statistical information is included in Table S1, Experiment 1.

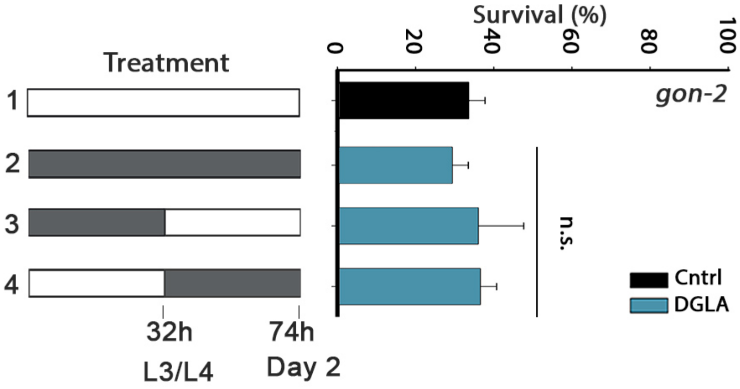

Supplementary Figure 3. The gonad is required for DGLA-dependent effects on heat shock survival in adulthood. Age-synchronized *gon-2* animals were grown on DGLA-supplemented plates, as indicated. Animals were subjected to heat shock (37 °C, 6 h) on day 2 of adulthood and survival was examined (>75 animals per data point, N>5). (n.s.) denotes non-significant.

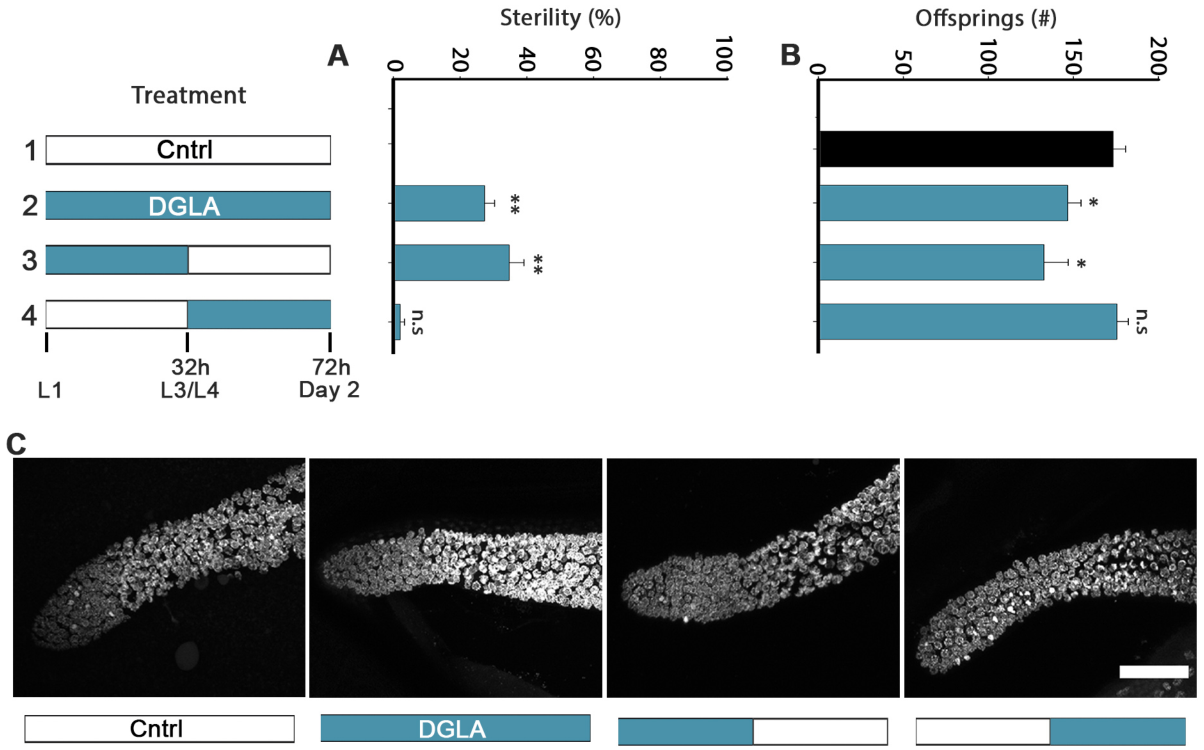

Supplementary Figure 4. DGLA have a mild effect on GSCs proliferation, brood size and sterility under our experimental conditions. (A) Percent of sterile animals scored in age-synchronized animals treated as indicated (>95 animals per data point, N>6). **(B)** Progeny numbers were scored in age-synchronized fertile animals treated as indicated (>15 animals per data point). **(C)** Representative images of germ cells from age-synchronized animals treated as indicated and stained with DAPI on day 2 of adulthood.

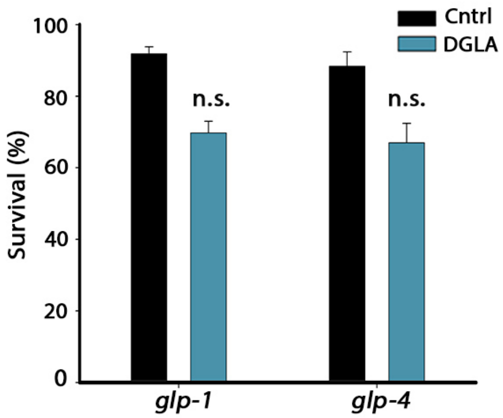

Supplementary Figure 5. DGLA-supplementation does not improve the thermo-resistance of germline mutants. Thermo-resistance was examined in age-synchronized *glp-1(e2141)* or *glp-4(bn2)* animals grown on control or DGLA-supplemented plates. Animals were subjected to a prolonged heat shock treatment (37°C, 6 h) and survival was assayed (>65 animals per data point, N>4). Data were compared to age-matched animals grown on control plates and no significant increase in survival was observed for animals grown on DGLA-supplemented plates.

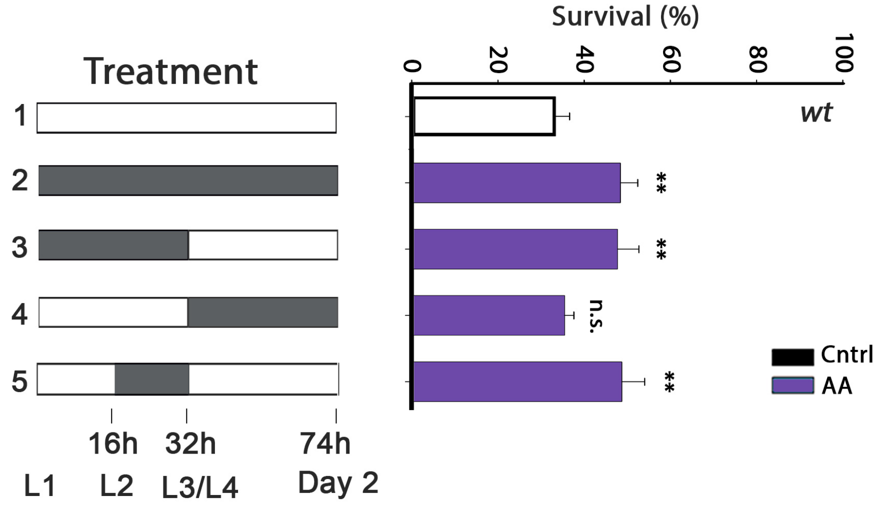

Supplementary Figure 6. Diet supplementation of AA during development modulates proteostasis of wild type animals in adulthood. Animals were subjected to a prolonged heat shock treatment (37°C, 6 h) on day 2 of adulthood and survival was assayed (>65 animals per data point, N>5). Data were compared to age-matched animals grown on control plates. (n.s.) denotes non-significant, (**) denotes *P*<0.01.

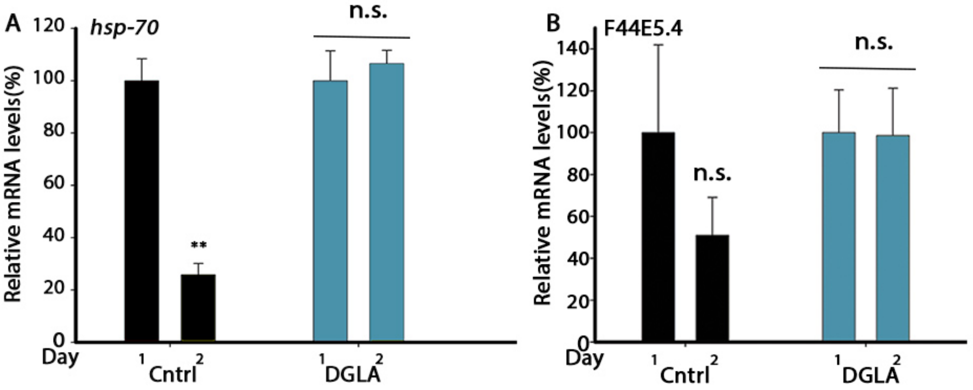

Supplementary Figure 7. DGLA supplementation resulted in maintenance of heat shock activation in adult animals. (A-B) Quantification of *hsp-70* (A) or *F44E5.4*(Hsp70) (B) mRNA levels from age-synchronized animals grown on control or DGLA-supplemented plates following heat shock (90 min at 37°C). The data presented are normalized to treated animals on day 1 of adulthood (N>4). Data were compared to treated animals on day 1 of adulthood. (n.s.) denotes non-significant, (**) denotes *P*<0.01.

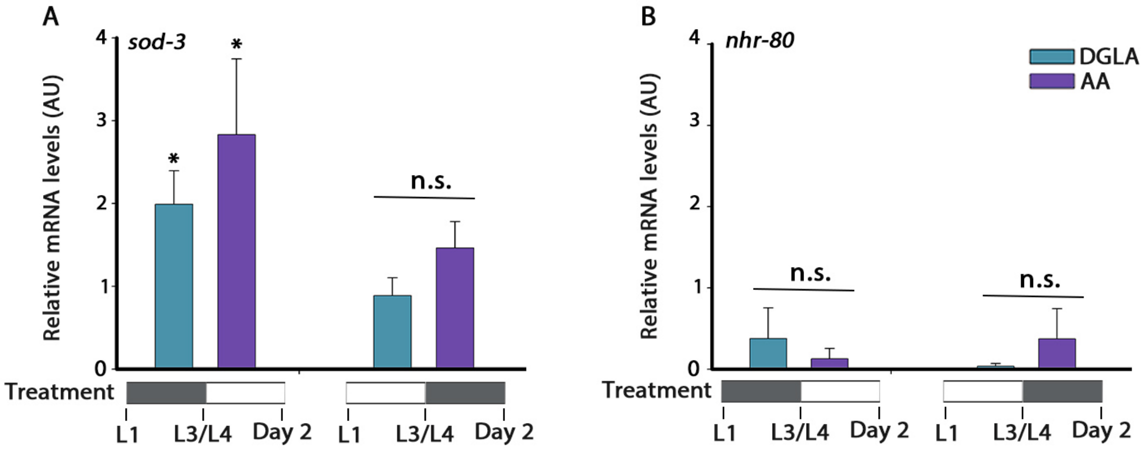

Supplementary Figure 8. DGLA and AA supplementation during development results in partial activation of the gonadal longevity pathway. (**A-B**) Quantification of *sod-8* (A) or *nhr-80* (B) mRNA levels from age-synchronized animals, treated as indicated. Data were normalized to those obtained with controls (N>4). Data were compared to age-matched animals grown on control plates. (n.s.) denotes non-significant, (*) denotes $P<0.05$.

2 Supplementary Table

Genotype	Treatment	Exp	Mean LS ± SEM (days)^	Events/Obs*	% of control	P Value vs. Control#
N2	Control	1	13.7±0.3	142(31)		
N2	DGLA		15±0.2	141(17)	111	<0.005
N2	DGLA -> Control		14.2±0.2	121(16)	104	n.s.
N2	Control -> DGLA		14±0.2	116(16)	102	n.s.
glp-1(e2141)	Control		16.9±0.3	150(16)	123	<0.001
N2	Control	2	13±0.4	124(49)		
N2	DGLA		15.2±0.3	170(53)	117	<0.001
N2	DGLA -> Control		13.5±0.5	125(64)	104	n.s.
N2	Control -> DGLA		13.8±0.4	105(30)	106	n.s.
N2	AA		14.4±0.3	200(62)	111	<0.005
N2	AA-> Control		14.4±0.5	129(59)	111	<0.005
N2	Control -> AA		14.3±0.3	120(54)	110	<0.05
glp-1(e2141)	Control		15.4±0.3	148(25)	118	<0.001
N2	Control	3	11.8±0.6	44(11)		
N2	DGLA		14.1±0.5	105(41)	119	<0.001
N2	DGLA -> Control		13.3±0.6	71(19)	113	n.s.
N2	Control -> DGLA		13.3±0.6	85(41)	113	n.s.
N2	AA		13.7±0.5	72(18)	116	<0.05
N2	AA-> Control		14.4±0.6	82(44)	122	<0.05
N2	Control -> AA		14.4±0.5	84(20)	122	<0.001
glp-1(e2141)	Control		16.1±0.3	175(53)	136	<0.001

Supplementary Table 1. DGLA or AA supplementation results in lifespan extension independent of the L2-L4 window. Lifespan of wild type (N2) or *glp-1(e2141)* animals grown on control or DGLA- or AA-supplemented plates. Animals were either grown continuously on control or treatment plates or shifted between treatments and control plates at the L3/L4 transition (>32 h), as indicated. ^Mean life span ± SEM calculated using Kaplan-Meier survival curves. *Animals that crawled off the plate, were contaminated or displayed a bagging phenotype (matricide due to internal hatching of embryos) were censored. #*P* values were determined using log-rank (Mantel-Cox) statistics.
